# Molecular Mechanisms and Targeted Intervention Strategies of Calcium Overload in Ischemic Stroke

**DOI:** 10.3390/ijms27052279

**Published:** 2026-02-28

**Authors:** Yuwei Jiang, Guijun Wang, Shengming Jiang, Youjun Wang, Qi Tian, Mingchang Li

**Affiliations:** Department of Neurosurgery, Renmin Hospital of Wuhan University, Wuhan 430060, China; jiangyuwei@whu.edu.cn (Y.J.); wgjwhuhan@whu.edu.cn (G.W.);

**Keywords:** ischemic stroke, calcium overload, excitotoxicity, neuroprotection

## Abstract

Ischemic stroke is a leading cause of global neurological mortality and disability, characterized by a complex pathogenesis wherein calcium overload constitutes a pivotal mechanism in neuronal and glial cell death. Following ischemia and reperfusion, excitotoxicity triggered by excessive glutamate release activates NMDA receptors and voltage-dependent calcium channels, leading to a large accumulation of intracellular Ca^2+^. This calcium dyshomeostasis subsequently initiates a cascade of detrimental events, including mitochondrial dysfunction, endoplasmic reticulum stress, reactive oxygen species generation, and the activation of calcium-dependent enzymes (such as calpain and phospholipase A_2_), ultimately culminating in cellular apoptosis or necrosis. In addition, calcium signaling imbalance is closely related to various forms of programmed cell death, such as ferroptosis and necroptosis. Research on calcium overload extends beyond neurons, with investigations in microglia and astrocytes yielding significant mechanistic insights. Although targeted interventions, encompassing calcium channel blockers, NMDA receptor antagonists, mitochondrial calcium homeostasis regulators, and calmodulin/calmodulin kinase (CaM/CaMK) inhibitors, have demonstrated preclinical progress, their clinical translation remains constrained. Future investigations should prioritize elucidating the nuanced regulatory mechanisms of calcium signaling pathways, developing highly selective and low-toxicity calcium intervention drugs, and combining multi-target treatment strategies such as neuroprotection, anti-inflammation, and tissue repair to provide theoretical basis and new solutions for the precise treatment of ischemic stroke.

## 1. Introduction

Ischemic stroke (IS), ranking as the second leading cause of death and the third leading cause of disability worldwide, has become a major public health challenge with significant implications for human health and social development [1,2]. Epidemiological data show that IS accounts for approximately 87% of all stroke cases [3]. In 2021, the global burden of IS was substantial, with an estimated 7.8 million patients (95% uncertainty interval [UI]: 6,719,760–8,943,692) and an age-standardized incidence rate (ASIR) of 92.39 per 100,000 population (that is, there were 92.39 new cases per 100,000 population) [4]. Furthermore, direct medical costs attributable to IS exceed 80% of total stroke-related expenses [5]. The pathological features of this disease are manifested as interruption of cerebral blood flow caused by thrombosis, atherosclerosis or embolism, leading to ischemic and hypoxic damage to brain tissue. Clinically, this manifests as neurological deficits including hemiplegia, aphasia, and cognitive impairment [6,7]. With the intensification of population aging, the dual burden imposed by IS on healthcare and social welfare systems continues to escalate, underscoring the urgent need to explore its pathological mechanism and identify novel therapeutic targets.

Current therapeutic strategies for IS primarily aim to rapidly restore cerebral blood flow to limit brain tissue damage. Intravenous thrombolysis, notably with recombinant tissue plasminogen activator (t-PA), represents a treatment method recommended by guidelines to dissolve the thrombus blocking the blood vessel and restore blood flow [8]. Despite its widespread clinical application, t-PA therapy is constrained by a narrow therapeutic time window, an increased bleeding risk upon time-window extension, and limited efficacy in large vessel occlusion [9]. Endovascular thrombectomy, an alternative interventional modality, physically removes clots and is particularly indicated for large vessel occlusion. However, this method also has its limitations such as heightened procedural risks in narrower vessels, insufficient evidence for central vessel occlusions, and no significant mortality reduction in large core infarctions. Although combining thrombectomy with thrombolysis can enhance recanalization rates, clinical benefits remain confined to a subset of patients [10].

Calcium overload has been identified as a central mechanism in cerebral ischemia–reperfusion injury during IS pathogenesis [11]. Under physiological conditions, Ca^2+^ serves as a fundamental regulatory factor for cellular functions and a crucial second messenger, thereby maintaining systemic homeostasis. As a crucial second messenger within cells, it participates in the neural transmission process, stabilizes the cell membrane potential and regulates the excitability of neurons [12,13]. Furthermore, Ca^2+^ mediates depolarization signal transduction, thereby influencing cytoskeletal reorganization, cell migration, and even immune escape. Its role is particularly critical in the nervous system, where Ca^2+^ facilitates neural signal transduction and neurotransmitter release through voltage-gated calcium channels(VGCCs), regulates mitochondrial ATP synthesis to sustain energy metabolism, and affects the expression of genes related to neural plasticity and cell survival through CaM pathways [14]. Another essential function involves intracellular Ca^2+^ homeostasis, which is precisely maintained by calcium buffering systems and specific ion channels such as Transient Receptor Potential Vanilloid 4 (TRPV4) [15]. Extracellular Ca^2+^ can also enter cells through plasma membrane channels and participate in regulatory processes under physiological and pathological conditions [16]. During the pathological process of IS, ischemia-induced Calcium overload disrupts this homeostasis, initiating cytotoxic cascades through protease activation and free radical generation, ultimately resulting in irreversible damage to neurons [17]. Consequently, targeting Calcium overload is beneficial for reversing or alleviating IS-related damage. Clinical evidence indicates that calcium homeostasis imbalance is closely related to the progression of IS, serum ionic calcium levels not only have a significant correlation with the severity of the disease (NIHSS score), but also affect infarction volume [18]. Notably, the relationship between calcium levels and prognosis exhibits a dual nature: while some studies associate higher serum calcium with improved outcomes [19,20], there is also evidence suggesting that calcium overload may worsen the recovery of neurological function by promoting mitochondrial dysfunction and other pathways [21]. This apparent contradiction highlights the necessity for further investigation into the dynamic regulatory networks governing calcium signaling in IS.

Based on the current understanding of IS pathogenesis, from calcium signal transduction at the microscopic level to the expansion of infarction foci at the macroscopic level, calcium homeostasis imbalance runs through all links of the ischemic cascade response. Animal experiments have confirmed that calcium-regulating drugs (such as Gen) can improve neurological function by reducing infarct area by approximately 34% [22]. These findings offer promising translational implications. A systematic elucidation of the spatiotemporal dynamics and underlying molecular mechanism of calcium overload is therefore essential, not only for informing neuroprotective strategies and developing precise prognostic frameworks, but also for overcoming existing therapeutic limitations.

## 2. Calcium Homeostasis and Calcium Overload

### 2.1. Maintenance of Normal Calcium Homeostasis

Under normal physiological conditions, neuronal Ca^2+^ concentrations are maintained in a tightly regulated dynamic balance. A substantial gradient exists between the cytoplasmic Ca^2+^ level (approximately 100 nM) and the concentrations found in the extracellular area (about 1.2 mM) as well as within intracellular reservoirs such as the endoplasmic reticulum (ER) and mitochondria (about 0.5–1 mM). This steady state depends on multiple mechanisms [23,24,25]: Primary regulation is achieved through the integrated activity of calcium channels and pumps. VGCCs, receptor-gated channels (such as NMDARs), ER calcium release channels (IP_3_R, RyR), and calcium pumps (SERCA, PMCA) collectively govern Ca^2+^ influx and efflux to maintain the concentration gradient. Furthermore, mitochondria regulate calcium uptake and release through MCU (mitochondrial calcium uniporter) and NCLX (Na^+^/Ca^2+^ exchanger), while the ER stores calcium through SERCA pumps, and IP_3_R and RyR mediate calcium release. In addition, calcium-binding proteins such as CaM further consolidate the homeostatic system by buffering free Ca^2+^ in the cytoplasm, thereby preventing local excessive concentration [14].

### 2.2. The Functional Characteristics of Ca^2+^

In neuronal cells, Ca^2+^ enters through VGCCs or membrane receptors, thereby triggering neurotransmitter release from synaptic vesicles, a fundamental process underlying neural transmission [13]. Concurrently, Ca^2+^ influx via VGCCs stabilizes membrane potential and modulates neuronal excitability, thereby influencing nerve impulse propagation [26]. In specific neurons (such as hippocampal neurons), Ca^2+^ signals regulate synaptic plasticity by activating calcium-dependent proteins including CaM-dependent protein kinase II (CaMKII), and participate in the learning and memory processes [27,28]. During development, calcium influx activates downstream pathways, guiding neuronal differentiation [29,30].

Under physiological conditions, Ca^2+^ signaling in astrocytes is primarily governed by GPCRs (e.g., purinergic receptors), which activate the phospholipase C–IP_3_R pathway and trigger Ca^2+^ release from the ER. Additional regulatory mechanisms include store-operated Ca^2+^ entry (SOCE) mediated by STIM1/Orai1 and the involvement of TRP channels. These finely tuned Ca^2+^ oscillations are indispensable for cerebral homeostasis; they underpin the release and reuptake of gliotransmitters such as ATP and glutamate, modulate synaptic plasticity and neurotransmission efficacy, and are central to neurovascular coupling—the process that dynamically adjusts local cerebral blood flow to meet neuronal metabolic demand. Moreover, these signals directly support the structural and functional integrity of the blood–brain barrier (BBB), modulate neurotransmission [31], and indirectly regulate neuronal activity [32,33,34].

In microglia, Ca^2+^ signaling primarily functions as a regulator of their sentinel activity within the CNS. Transduction occurs via P2X7/P2Y purinergic receptors, CRAC channels (predominantly Orai1), and TRP channels. Transient, tightly regulated Ca^2+^ elevations drive dynamic process extension, directional migration, and chemotactic responses to minute concentrations of damage-associated signals such as ATP. These calcium events constitute the cellular basis for continuous parenchymal surveillance, efficient clearance of microdebris, and maintenance of immune quiescence [35]. In summary, Ca^2+^ orchestrate neurotransmission, excitability, and pathological responses in neurons, whereas in glial cells they are engaged in neuromodulation and inflammatory control.

**Figure 1:**
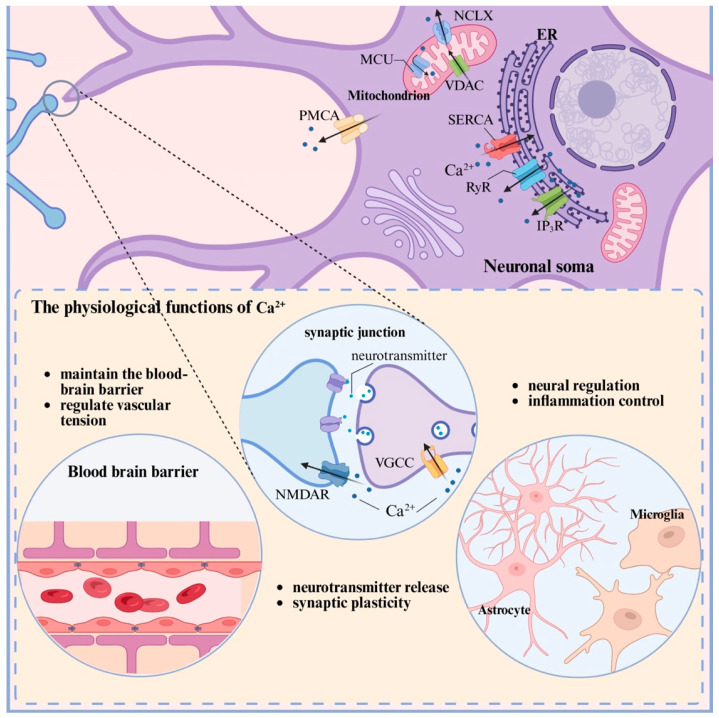
The functions of Ca^2+^. Under physiological conditions, precise Ca^2+^ homeostasis is maintained by channels, pumps, and buffers. Ca^2+^ signals regulate critical functions: neurotransmitter release and synaptic plasticity in neurons; neuroregulation and inflammation control in glial cells; and blood–brain barrier (BBB) integrity and vascular tone in endothelial cells.

As integral components of the BBB, cerebral microvascular endothelial cells rely on Ca^2+^ signaling to precisely regulate barrier integrity, permeability, and neurovascular coupling. Serving as a versatile second messenger, calcium integrates mechanical stimuli (e.g., shear stress sensed via Piezo1 channels) and circulating humoral factors. Through pathways involving VGCC subunits (e.g., Cavβ3) and GPCRs, intracellular Ca^2+^ dynamics modulate the phosphorylation status of tight junction proteins (e.g., Claudin-5, Occludin) and actin cytoskeletal remodeling. These events dynamically sustain selective permeability and effectively exclude neurotoxic substances from the brain parenchyma [36,37,38,39]. Elevated endothelial Ca^2+^ also promotes the release of vasodilatory mediators such as nitric oxide (NO) and prostaglandins, thereby fine-tuning vascular tone and ensuring adequate oxygenation and neurovascular coupling within the cerebral microcirculation [40,41]. Furthermore, Ca^2+^ signals mediate neurotransmitter-evoked responses in endothelial cells; for instance, GABA receptor activation elicits a biphasic Ca^2+^ elevation via IP_3_-dependent ER release and subsequent SOCE, contributing to signal integration and neuromodulatory functions in the endothelium [42]. Pericytes, which constitute another essential component of the microvascular wall, exhibit Ca^2+^ signals that coordinate their contractile and relaxant states under physiological conditions. This signaling directly governs capillary tone and local cerebral blood flow, thereby exerting a critical influence on neurovascular coupling and microcirculatory stability [43] (Figure 1).

### 2.3. Calcium Overload Mechanism

Calcium overload refers to a state characterized by disrupted intracellular calcium homeostasis, wherein Ca^2+^ accumulation surpasses physiological regulatory capacity and subsequently initiates multiple damage networks. This overload originates from both transmembrane Ca^2+^ influx and abnormal release of intracellular calcium reservoirs under ischemic conditions. The ensuing cytotoxicity is mediated by the activation of various Ca^2+^-dependent enzymes, including calpain, phospholipase A_2_ (PLA_2_), and endonucleases [23].

Meanwhile, mitochondrial function becomes critically impaired through the synergistic effects of calcium overload and ROS. This interaction promotes the opening of mitochondrial permeability transition pores (mPTP), leading to the collapse of mitochondrial membrane potential and cessation of ATP synthesis. The resulting energy crisis is compounded by cytochrome c release, which activates the apoptotic pathway, while dysfunction of the MCU and voltage-dependent anion channel (VDAC) further exacerbate mitochondrial calcium accumulation [44].

Furthermore, calcium overload induces ROS bursts via mitochondrial electron leakage and NADPH oxidase (NOX) activation. These ROS, in turn, inhibit antioxidant systems such as glutathione, thereby establishing a self-perpetuating vicious cycle of damage [14,45].

Other mechanisms can significantly amplify this injury. For instance, the excitotoxicity of glutamate and the effect of transient receptor potential (TRP) channels can also amplify the damage. Specifically, the coupling of extrapsynaptic N-methyl-D-aspartate receptor (esNMDAR) with TRPM2 can prolong calcium influx [46]. Section 3 provides a specific summary of the most salient mechanisms and pathological consequences of calcium overload in IS (Table 1).

## 3. The Pathological Role of Calcium Overload in Ischemic Stroke

### 3.1. Calcium Overload Mediates Neuronal Apoptosis

Calcium overload serves as a direct trigger for neuronal apoptosis by initiating multiple enzymatic cascades. Following IS, the pathologically elevated of intracellular Ca^2+^ concentration first activates the calcium-dependent protease calpain. This enzyme hydrolyzes cytoskeletal proteins and compromises BBB integrity by disrupting the tight junction protein ZO-1, consequently resulting in the loss of neuronal structural integrity [23,47]. Concurrently, calcium overload can induce the Ca^2+^/CAM-CAMKII-DRP1 signaling axis: upon binding Ca^2+^, CaM activates CaMKII, which subsequently promotes the phosphorylation of mitochondrial fission protein DRP1. This cascade leads to excessive mitochondrial fission and disruption of energy metabolism, ultimately driving neuronal apoptosis [48,49]. Furthermore, calcium overload specifically upregulates and promotes polymerization of TLK2 kinase, an event that induces nuclear membrane rupture and abnormal entry of neurons into the mitotic cycle (mitotic disaster), further amplifying the apoptotic effect [50]. These processes are intimately linked to excitotoxic mechanisms. Specifically, coupling between esNMDAR and TRPM2 channels prolongs calcium influx, while downregulation of astrocytic glutamate transporter 1 (GLT-1) impairs synaptic glutamate clearance. The abnormally activated NMDAR-CaMKIIα pathway promotes the pro-apoptotic signal JNK to dominate the downstream death program [46]. Notably, the calcium influx mediated by the NMDA receptor GluN2B subtype exhibits threefold greater intensity than that of GluN2A, substantially exacerbating mitochondrial calcium overload and accelerating apoptosis [51,52,53].

### 3.2. Calcium Overload Induces Oxidative Stress and Free Radical Burst

Calcium overload and oxidative stress interact through a self-reinforcing vicious cycle that significantly amplifies neuronal injury. Mitochondrial calcium overload directly inhibits the activities of respiratory chain complexes I and III, and cytochrome c oxidase (COX), while simultaneously triggering reactive oxygen species (ROS) burst through electron leakage and NOX activation. The resulting oxidative stress subsequently suppresses antioxidant systems such as glutathione, thereby establishing a positive feedback damage loop that perpetuates cellular damage [14,45,54,55].

This destructive interplay is regulated by multiple signaling pathways: After the activation of the Wnt/Ca^2+^ pathway, the calcium-dependent protein calpain and phosphorylated CaMKII synergistically promote ROS generation and upregulate the expression of the apoptotic executive protein caspase-3 [22]. Meanwhile, the dysregulation of the PI3K/AKT pathway causes calcium overload to activate PLA_2_ and inducible nitric oxide synthase (iNOS), consequently promoting the release of ROS/RNS and exacerbating mitochondrial dysfunction and oxidative damage [56].

### 3.3. Calcium Overload Leads to Mitochondrial Collapse and Energy Depletion

Mitochondrial dysfunction represents the central pathological outcome of calcium overload. The initial disruption originates from impaired gating of the MCU complex: under pathological conditions, MICU1 subunit depolymerization and detachment from the MCU pore reduces the calcium influx threshold to 3 μM and accelerates calcium overload. Simultaneously, the NCLX exhibits markedly reduced calcium excretion efficiency due to insufficient phosphorylation of PKA, resulting in the collapse of calcium homeostasis [57]. Furthermore, calcium overload directly induces the opening of mPTP, which precipitates membrane potential collapse, cytochrome c release, and interruption of ATP synthesis, subsequently activating mitochondrial-dependent apoptotic pathways [44,55]. This process triggers a vicious cycle of energy metabolism wherein calcium overload inhibits oxidative phosphorylation and reduces ATP production, while ATP depletion deactivates the calcium pump (PMCA/SERCA), ultimately forming an irreversible damage chain reaction of “calcium overload → ATP depletion → pump deactivation → calcium overload” [57,58,59,60].

### 3.4. Calcium-Dependent Protease Activation and Cytoskeletal Disintegration

As a principal effector of calcium overload, the abnormal activation of the calpain system directly compromises cellular structural integrity. A sudden increase in calcium concentration induces calpain conversion from its inactive precursor to an active state, which subsequently cleaves cytoskeletal proteins and disrupts neuronal architecture. In vascular endothelial cells, calpain degrades the BBB tight junction protein ZO-1, increasing permeability and exacerbating cerebral edema. In addition, calpain proteolyzes neuroprotective proteins including TRPC6 and TrkB, which inhibits their functions and promotes the excessive activation of NMDA receptors. TRPM2 can also overactivation drives dysfunction of NVUs [61]. These collective mechanisms ultimately converge to induce neuronal death [23,47].

### 3.5. Calcium Overload Disrupts the Function of Non-Neuronal Cells

#### 3.5.1. Astrocytes

Ischemia–reperfusion injury triggers an energetic crisis, leading to excessive extracellular accumulation of glutamate and overactivation of metabotropic glutamate receptors (mGluRs) and P2Y receptors on astrocytes. This strongly amplifies IP_3_R-mediated Ca^2+^ release from the ER. Subsequent ER store depletion robustly activates SOCE via the STIM1/Orai1 pathway, driving sustained extracellular Ca^2+^ influx and generating pronounced, persistent global Ca^2+^ signals. Prolonged STIM1 activation also secondarily elevates intracellular Na^+^ concentrations, disrupting the Na^+^ gradient required for GLT-1 function [32].This Calcium overload induces reactive astrogliosis and causes GLT-1 to reverse its mode of operation—shifting from glutamate uptake to massive glutamate release—thereby directly exacerbating neuronal excitotoxicity. Concurrently, it promotes cytotoxic edema, triggers mitochondrial dysfunction, and amplifies neuroinflammation and oxidative stress via release of inflammatory mediators, culminating in blood–brain barrier (BBB) disruption and progressive conversion of the ischemic penumbra into irreversible infarction [32,33,34].

#### 3.5.2. Microglia

In the early phase of ischemia, high local concentrations of damage-associated molecular patterns (DAMPs), such as ATP released from injured cells, potently activate P2X7 receptors on microglia, eliciting robust, oscillatory Ca^2+^ waves. SOCE mediated by CRAC channels becomes persistently amplified, and Ca^2+^ homeostasis is profoundly dysregulated. During reperfusion, oxidative stress and pro-inflammatory signals (e.g., via TLR4 signaling) further exacerbate Ca^2+^ influx, and these Ca^2+^ dynamics become temporally synchronized with cortical spreading depolarization (CSD) waves, forming a self-reinforcing vicious cycle [35,62]. Sustained, pathological Ca^2+^ signaling drives microglial polarization toward a pro-inflammatory M1 phenotype, accompanied by massive release of TNF-α, IL-1β, and ROS, thereby aggravating neuroinflammation and oxidative damage. These aberrant Ca^2+^ waves, in coordination with CSD, propagate injury signals into the penumbra. Moreover, dysregulated Ca^2+^ signaling leads to excessive phagocytosis of still-viable synapses (synaptic stripping), disrupting neural network connectivity. Collectively, these processes compromise BBB integrity, facilitate infiltration of peripheral immune cells, and accelerate infarct progression.

#### 3.5.3. Vascular Endothelial Cells

During ischemia, ATP depletion and oxidative stress directly activate TRPM2 channels in endothelial cells, triggering Ca^2+^ influx. Upon reperfusion, restoration of blood flow generates aberrant shear stress, which strongly activates the mechanosensitive Piezo1 channel and induces even more pronounced Ca^2+^ entry, subsequently engaging the CaMKII/calpain pathway [38]. Calcium overload, via CaMKII/calpain signaling, directly degrades tight junction proteins (e.g., ZO-1, occludin), leading to structural disintegration of the BBB, a sharp increase in permeability, and life-threatening complications such as vasogenic cerebral edema and hemorrhagic transformation. Concurrently, endothelial cells undergo apoptosis and ferroptosis, release inflammatory mediators, and disrupt NVU coupling—substantially aggravating reperfusion injury and worsening clinical outcomes [37,38,39].

#### 3.5.4. Pericytes

Ischemia induces elevated intracellular Ca^2+^ concentrations in pericytes, lead ing to aberrant activation of the Ca^2+^-dependent chloride channel TMEM16A (also known as ANO1). This activation elicits membrane depolarization, which in turn opens VGCCs, establishing a positive feedback loop—“Ca^2+^ influx → Cl^−^ efflux → further depolarization → enhanced Ca^2+^ influx”—resulting in tonic, irreversible pericyte contraction. Such sustained contraction directly compresses and collapses capillary lumens, representing a core mechanism underlying the “no-reflow” phenomenon even after successful recanalization of large vessels. This contractile rigor persists even after pericyte death, chronically impeding restoration of blood flow and tissue oxygenation, thereby rendering the penumbra incapable of effective reperfusion and driving its progression to infarction [43,63].

### 3.6. Cascade Damage Caused by ER Calcium Overload

The disruption of ER calcium homeostasis represents a critical mechanism of transorganelle injury in IS. Impaired SERCA pump function results in cytoplasmic calcium accumulation and depletion of ER calcium reservoir, thereby activating the unfolded protein response (UPR). While initial UPR activation through the PERK-EIF2α and ATF6 pathways attempts to reestablish cellular equilibrium, persistent calcium overload converts this adaptive response into a pro-apoptotic mode that accelerates neuronal death [64,65,66]. Furthermore, ER calcium overload also increases calcium flow to mitochondria through mitochondrial-associated membranes (MAMs), triggering mitochondrial calcium overload, ROS burst and the release of apoptotic factors, and forming the ER-mitochondrial apoptotic axis [67,68].

**Figure 2:**
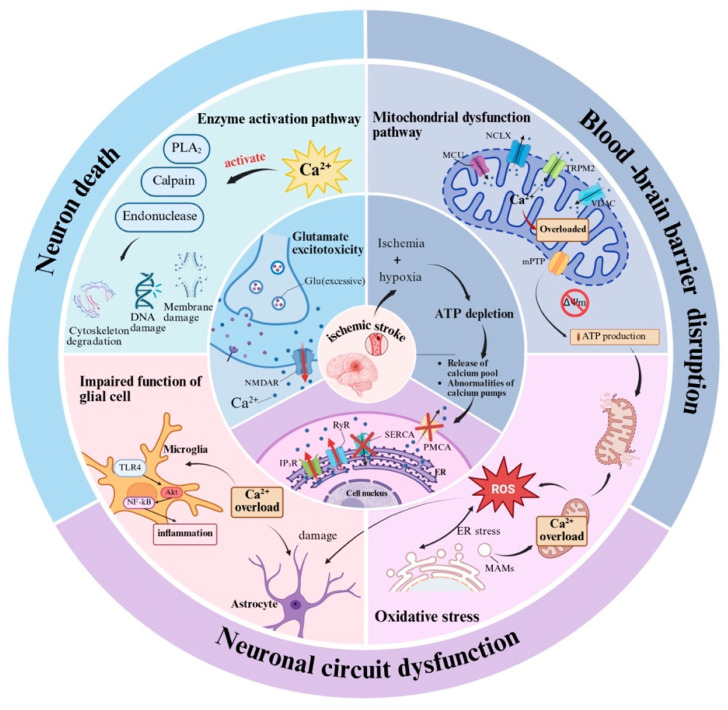
The mechanisms and pathological role of calcium overload in IS. This diagram delineates the vicious cycle of calcium overload triggered by cerebral ischemia and its pivotal role in driving neuronal injury and death: Ischemia-induced ATP depletion and glutamate excitotoxicity initiate calcium overload. This triggers a destructive network: 1. Enzyme activation (e.g., calpain) damages cellular structures. 2. Mitochondrial dysfunction causes energy collapse and apoptosis. 3. Oxidative stress and 4. Neuroinflammation create vicious cycles that amplify the initial injury, leading to irreversible cell death.

Consequently, calcium overload functions as the core hub of ischemic brain injury, driving irreversible neuronal damage through multi-level pathological networks such as enzymatic cascade activation (calpain, TLK2), CaMKII-DRP1 signaling axis, mitochondrial energy breakdown, oxidative stress burst, glial cell dysfunction, and ER-mitochondrial apoptotic pathways. These collective insights position calcium overload as a pivotal target for developing precise therapeutic interventions (Figure 2).

## 4. The Spatiotemporal Evolution of Calcium Overload

### 4.1. The Relationship Between Calcium Overload Mechanisms and Ischemic Du Ration Dynamics

In IS, calcium overload is not an instantaneous event but rather a dynamic, progressive cascade tightly coupled to the duration of ischemia. In the early phase (seconds to minutes) of ischemia, abrupt interruption of blood flow leads to rapid ATP depletion, failure of Na^+^/K^+^-ATPase, and subsequent membrane depolarization, which activates VGCCs and glutamate receptors—particularly esNMDARs—resulting in massive extracellular Ca^2+^ influx [69]. This initial Ca^2+^ surge activates downstream phospholipases and proteases, initiating early injury cascades. As ischemia prolongs (minutes to hours), ER Ca^2+^ stores become depleted, and sustained amplification of cytosolic Ca^2+^ occurs via STIM1/Orai1-mediated SOCE. Concurrently, mitochondria take up substantial Ca^2+^ through the MCU, leading to matrix Calcium overload, collapse of mitochondrial membrane potential, burst of ROS, and eventual opening of the mPTP, which releases pro-apoptotic factors. During the reperfusion phase (minutes to hours after blood flow recovery), restoration of oxygen supply triggers an “oxidative burst,” further exacerbating Ca^2+^ influx and mitochondrial Calcium overload, thereby establishing a self-amplifying vicious cycle that drives delayed neuronal death [70]. This dynamic continuum illustrates that calcium overload evolves from an initial triggering signal in early ischemia into a central executor that amplifies injury and dictates cell fate during reperfusion. Once ischemic duration exceeds a critical window (typically 6–8 h), the damage driven by calcium overload progresses from reversible penumbral tissue to irreversible infarction in the ischemic core [71].

### 4.2. Distinct Profiles of Calcium Overload in Ischemic Core Versus Penumbra: Implications for Tissue Fate

The ischemic core and the ischemic penumbra exhibit fundamental differences in cere bral blood flow, cellular metabolic status, and calcium overload characteristics—distinctions that directly determine their respective fates and responsiveness to therapeutic intervention. In the ischemic core, cerebral blood flow is severely reduced (typically <10–20 mL/100 g/min), leading to rapid and irreversible energy failure. Under these conditions, calcium overload occurs precipitously and with high magnitude: abrupt membrane depolarization triggers a torrential Ca^2+^ influx through VGCCs and NMDARs, while excessive mitochondrial Ca^2+^ uptake via MCU rapidly induces mPTP opening, massive ROS generation, and cytochrome c release, culminating in predominantly necrotic cell death within minutes [70]. The microenvironment of the core is intensely pro-inflammatory, exemplified by microglial upregulation of LGALS3, which exacerbates inflammation and fuels glycolysis-dependent injury amplification [71]. In contrast, the ischemic penumbra retains residual perfusion (20–50 mL/100 g/min) and partial energy metabolism, rendering cellular injury progression relatively slower and, within a definable time window, reversible. Calcium overload in this region manifests as intermittent, low-amplitude Ca^2+^ transients or oscillations rather than sustained supraphysiological elevations [72]. Penumbral cells can mount certain endogenous protective responses—such as upregulation of antioxidant enzymes—to counteract oxidative stress and inflammation downstream of calcium overload. Microglia in this zone may assume distinct functional phenotypes, for instance expressing PIK3IP1, which confers enhanced anti-inflammatory and reparative capacity [71].

Nevertheless, if ischemia persists or reperfusion injury is inadequately managed, calcium overload-driven apoptotic signaling within the penumbra (e.g., sustained mitochondrial damage, calpain activation) and inflammatory cascades progressively intensify. This ultimately leads to neuronal death in the penumbra, which gradually becomes incorporated into the expanding infarct core, thereby increasing total infarct volume [70]. Accordingly, the key to salvaging the penumbra lies in achieving timely, effective reperfusion within the therapeutic window, combined with neuroprotective agents that precisely modulate specific nodes of the calcium overload cascade, thereby intercepting its progression toward irreversible injury.

## 5. Intervention Strategies for Calcium Overload

### 5.1. Intervention Strategies Targeting Enzymes

(1) Calpain inhibitors. Pharmacological inhibition of calpain activity, exemplified by agents such as calpeptin, alleviates neuroinflammatory responses while simultaneously promoting vascular regeneration and blood supply in ischemic areas [73]. Such intervention mitigates BBB dysfunction and reduces neuronal damage, including neuronal protection in the hippocampus [74]. Although calpain-targeted intervention holds broad therapeutic potential, no calpain-specific inhibitors have yet received clinical approval.
(2) Intervention in the CaM/CaMKII/DRP1 pathway. The calcium chelator BAPTA-AM effectively counteracts the elevated intracellular Ca^2+^ concentration induced by methylmercury (MeHg). By blocking this pathway, BAPTA-AM restores mitochondrial dynamic balance and attenuates apoptotic cell death [49].
(3) TLK2 kinase inhibition. Targeting TLK2 kinase with small-molecule inhibitors such as promethazine (PMZ) can reduce calcium overload-induced neuronal death by suppressing TLK2 kinase activity. Corresponding therapeutic effects can also be achieved by inhibiting its downstream effectors, notably through myosin IIA inhibitors. For instance, blebbistatin can inhibit myosin IIA function, alleviate TLK2-mediated nuclear membrane rupture, or interfere with the binding of LC8 to TLK2, which can also weaken the formation of TLK2-Myosin IIA complexes, thereby inhibiting cell death [50].
(4) Modulation of the Wnt/Ca^2+^ signaling pathway. Genistein (Gen) can block the calcium release pathway and reduce calcium overload by downregulating the expression of Wnt5a, Frizzled-2, p-CaMKII and IP_3_R. Additionally, Gen can up-regulate the expression of SOD1/SOD2 and lower the levels of ROS and MDA, resulting in reduced oxidative damage [22].
(5) PI3K/AKT pathway activation. The compound 6′-O-caffeyl arbutin (CA) can activate the PI3K/AKT pathway, upregulate the anti-apoptotic protein Bcl-2, inhibit Bax expression, and reduce the ratio of Bax/Bcl-2. This regulation decreases the activation of CASP3; concurrently, CA can reduce the phosphorylation of p-IκBα and P65, decrease the release of inflammatory factors, and indirectly alleviate the abnormal opening of calcium channels [56].

Although there are various targeting angles and methods for the aforementioned enzymes related to calcium overload, there are still many unsolved problems and areas to be explored. For example, the enzymatic characteristics and physiological functions of mitochondrial calpain, such as calpain-13, remain poorly characterized [75], and significant translational barriers persist. This is exemplified by the current absence of clinically approved calpain-specific inhibitors, compounded by the fact that different calpain subtypes exert distinct effects, thus requiring precise subtype regulation [76]. Meanwhile, most drugs or compounds remain confined to cellular or animal models, with substantial progress still required before their clinical application for mitigating calcium overload in IS can be realized.

### 5.2. Intervention Strategies Targeting Mitochondria

Several targeted intervention strategies have been developed to counteract mitochondrial calcium overload, aiming to preserve mitochondrial integrity and improve the prognosis of stroke. Targeting calcium influx, the potent MCU channel blocker Ru265 (Kd = 0.2 nM) can effectively reduce mitochondrial Ca^2+^ internal flow [77,78]. Similarly, 2-APB, an IP_3_R antagonist, along with GSK3β inhibitors such as SB216763 can inhibit the IP_3_R-GRP75-VDAC1 complex, thereby blocking the flow of Ca^2+^ from ER to mitochondria [79,80]. Curcumin, as a natural regulator, can lower the mitochondrial calcium levels and prevent mitochondrial swelling caused by calcium overload [81]. An alternative approach focuses on enhancing calcium excretion. For instance, Bay 60-7550 can inhibit PDE2 → increase cAMP → activate PKA → phosphorylate the NCLX-S258 site, thereby enhancing the excretion efficiency of Ca^2+^ [82]. To improve targeting efficiency, nanotechnological delivery systems are being explored. These include TAT peptides for membrane penetration and mitochondrial enzyme targeting, and GYR peptides to bind to the transferrin receptors of diseased NVUs, achieving precise enrichment [57].

### 5.3. Intervention Strategies for ER

Intervention strategies for ER calcium overload mainly include regulating SERCA function and inhibiting ER stress and UPR. On the one hand, enhancing SERCA activity is a core strategy for alleviating calcium overload. For instance, distal ischemic postconditioning (RIPostC) reduces ER stress and calcium overload by upregulating SERCA2 expression, thereby providing neuroprotection. Similarly, SERCA2a gene therapy has also shown effects in reducing cytoplasmic calcium overload and improving pathological phenotypes in animal models [60,83]. On the other hand, pharmacological inhibition of ER stress and UPR is equally crucial. For instance, neuroserpin alleviates ischemic brain injury and prevents calcium imbalance and apoptosis by suppressing ER stress signaling molecules such as PERK-EIF2α [84]. Additionally, vitamin D_3_ (VitD_3_) exerts a protective effect by acting at the intersection of ER stress and ferroptosis [85].

### 5.4. Targeting the Glutamate System

The interventions focusing on the glutamate system encompass three primary approaches. First, inhibiting the excessive activation of NMDA receptors: Telaprevir can reduce the phosphorylation of GluN2B by inhibiting the activity of MALT1 protease, thereby lowering calcium influx and necrosis and apoptosis [86]. Similarly, memantine acts as a selective antagonist of esNMDAR that blocks calcium influx mediated by GluN2B [87]. Second, enhancing glutamic acid clearance: SHPL-49 upregulates the astrocyte glutamte transporter GLT-1 to promote glutamate reuptake [46]. Alternatively, small-sized graphene oxide (s-GO) alleviates excitotoxicity by reducing presynaptic glutamate release [88]. Third, directly modulating calcium balance: Epigallocatechin gallate(EGCG) upregulates the calcium-binding protein hippocalcin to inhibit the Bax/caspase-3 pathway, thereby alleviating glutamate-induced neuronal death [89]. Retinoic acid reduces intracellular calcium concentration by promoting parvalbumin expression [90]. A major limitation in current excitotoxicity research is the high clinical failure rate, attributable to issues of non-specificity, severe side effects [91,92], pathway redundancy caused by mechanisms complexity and drug delivery challenges. Future research should focus on multi-target therapies or the use of nanotechnology to enhance delivery efficiency and other aspects, in order to overcome these deficiencies and improve therapeutic effects.

### 5.5. Targeting TRP Channels

TRP channels, functioning as non-selective cation channels, participate in the pathological process of IS by mediating Ca^2+^ influx. This large channel family includes many categories, such as TRPM, TRPC, and TRPV. For key TRP subtypes, targeted regulation can also be carried out: TRPM2 inhibitors (such as ACA, TAT-M2 peptide, and clotrimazole) can alleviate calcium overload by blocking the ADPR binding domain or inhibiting channel activity [37,93]. Kuanxiong Aerosol (KXA) downregulates the expression of TRPV1, thereby improving mitochondrial function [94]. Furthermore, hyperforin extract and resveratrol can exert a dual protective effect by inhibiting TRPC6 degradation or activating the CREB pathway [37,95]. In addition, the TRPV4 inhibitor GsMTx4 and the TRPM4 antagonist glibenpiride can alleviate brain edema [96,97]. Nevertheless, Obstacles in TRP-targeted therapy include a scarcity of subtype-specific drugs [98]. Persistent challenges, particularly off-target side effects (such as abnormal neural excitability or immune dysfunction [99,100]) and insufficient delivery efficiency, necessitate integrated approaches combining structural analysis techniques with nanocarrier optimization strategies.

### 5.6. Calcium Channel Blockers (CCBs)

Abnormal activation of VGCCs (such as L-type and T-type) is an important source of calcium overload after ischemic injury [14,101]. T-type CCBs such as mibedil and ML218 inhibit TRPV1-mediated calcium influx, which subsequently reduces CaMKII activity and DRP1 phosphorylation, thereby preserving mitochondrial function [48,94,102]. Likewise, the L-type CCB nimodipine alleviates vasoconstriction and improves cerebral blood flow by antagonizing the CaV1.2 channel [103,104]. However, traditional CCBs have limitations such as insufficient target specificity (such as amlodipine), low selectivity for neuronal Ca^2+^ channels, which may limit therapeutic efficacy [105,106], alongside hemodynamic risks wherein excessive hypotension may aggravate cerebral ischemia, especially in patients with hypoperfusion [104]. In the future, the efficacy and safety can be enhanced by developing multi-target drugs (combined with antioxidants or anti-inflammatory agents) or nanotechnology-based delivery systems [107].

### 5.7. Other Intervention Targets

Beyond the above-mentioned mechanisms, several emerging strategies confer neuroprotection by regulating calcium homeostasis. For instance, brevetoxin-2 can influence NMDAR-mediated calcium signaling through sodium channel regulation [108]. Tetrapod framework nucleic acid (tFNA) similarly protects astrocytes by suppressing calcium overload and ROS generation during oxygen-glucose deprivation/reoxygenation (OGD/R) [51]. Mitochondrial Na^+^/Ca^2+^ exchange blockers (such as 4,1-benzothiazoles) and insulin pretreatment can reduce calcium influx and mitochondrial depolarization [109,110,111]. The chemokine receptor CX3CR1 alleviates neurotoxicity by inhibiting calcium influx [52]. CRAC channel inhibitors (such as CM-EM-137) antagonize microglial activation by blocking SOCE [111,112]. The SOCE mechanism regulated by the STIM1/Orai1 complex is related to autophagy regulation, revealing a novel therapeutic direction [53]. Collectively, these multi-pathway intervention strategies form a calcium overload regulatory network, opening up diverse target options for IS treatment (Table 2).

### 5.8. The Predicament of Clinical Transformation

To clearly demonstrate the current status of transformation, we have drawn a table to summarize the preclinical effects and clinical outcomes of the main calcium-targeted drugs (Table 3).

Despite the identification of numerous molecular targets in preclinical studies, neuroprotec tive drugs have consistently failed in clinical trials. This does not invalidate the “calcium overload” hypothesis, but rather reflects a systematic neglect of five critical translational dimensions.

Timing: Severe Mismatch of the Therapeutic Window

The excitotoxic cascade triggered by calcium overload evolves predominantly within minutes to one hour after ischemia—the golden window for neuroprotective intervention. In clinical reality, however, substantial delays from symptom onset to hospital admission, diagnosis, and drug administration are inevitable. Early landmark trials, such as the INWEST study with nimodipine, administered treatment up to 12 h post-onset, completely missing this critical window of molecular events and thus failing to reverse neuronal death in the ischemic core [113]. Even the aggressively designed FAST-MAG trial, which administered prehospital intravenous magnesium sulfate at a median of 45 min, showed no functional benefit, demonstrating that ultra-early administration alone is insufficient when the drug itself has inherent limitations (e.g., poor BBB penetration) [132].

Dose: narrow therapeutic window and dose-limiting toxicity

Neuroprotective doses identified in animal models often cannot be achieved in humans due to severe adverse effects. Many calcium modulators exhibit a U-shaped dose–response curve. For example, high doses of NMDA receptor antagonists (e.g., Selfotel, Aptiganel) provoked unacceptable neuropsychiatric toxicities—hallucinations, agitation, coma—in clinical trials, forcing dose reductions that resulted in brain concentrations insufficient to effectively block pathological Ca^2+^ influx [133]. Magnesium sulfate trials faced analogous dilemmas: high doses risked systemic hypotension and respiratory depression, whereas low doses lacked efficacy [134].

Specificity: disruption of physiological function and complex drug interactions

Early-generation agents lacked cellular or pathway specificity, blocking pathological pro cesses while simultaneously interfering with indispensable physiological Ca^2+^ signals. Broad-spectrum NMDA receptor antagonists indiscriminately inhibited both synaptic (pro-survival) and extrasynaptic (pro-death) NMDARs, potentially accelerating neuronal death [114]. More recent, ostensibly more specific compounds—such as Nerinetide (NA-1), designed to precisely disrupt the NMDAR–PSD95–nNOS death signaling complex—have encountered new obstacles. The ESCAPE-NA1 trial (2020) revealed that Nerinetide’s efficacy was completely abrogated by concomitant intravenous alteplase, as plasmin generated from tPA metabolism degrades this peptide drug in the bloodstream. This finding underscores the pharmacokinetic vulnerability of highly specific agents within complex clinical therapeutic regimens [118,135].

Drug delivery and BBB penetration: failure to reach the target

Even when a drug can be administered early, at sufficient dose, and with acceptable systemic safety, traversing an intact blood–brain barrier to reach ischemic brain parenchyma remains a formidable hurdle. Magnesium sulfate exemplifies this challenge: although intravenous infusion markedly elevates serum magnesium concentrations, the polar Mg^2+^ ion crosses the intact BBB extremely inefficiently, resulting in slow and modest increases in cerebrospinal fluid and brain parenchyma, far below the neuroprotective threshold during the acute phase [132,134]. Furthermore, post-ischemic microvascular “no-reflow”—partly attributable to sustained pericyte contraction—further impedes the delivery of blood-borne drugs to the critically endangered ischemic penumbra [136,137].

Side effects: systemic actions counterbalance local neuroprotection

Systemic side effects elicited by many calcium-modulating agents directly offset their potential neuroprotective benefits and may even exacerbate injury. The most salient example is the L-type CCB nimodipine. Its potent peripheral vasodilatory action induces systemic hypotension. Because vessels in the ischemic penumbra are already maximally dilated and passively dependent on systemic perfusion pressure, nimodipine-induced hypotension provokes a “steal phenomenon”—diverting blood flow from the low-resistance ischemic territory to drug-dilated normal vessels—thereby exacerbating penumbral ischemia. This mechanism was identified as a principal cause of failure in multiple clinical trials [104,138]. Likewise, the psychotomimetic effects of NMDA receptor antagonists complicate clinical management and compromise trial blinding [139,140].

**Figure 3:**
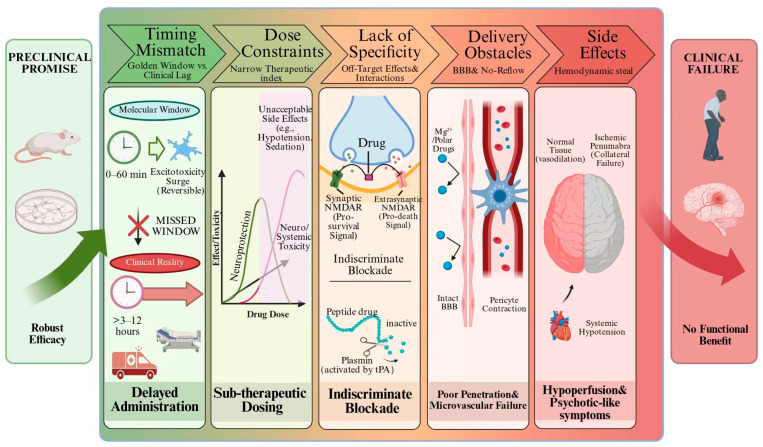
The translational gauntlet illustrating failures in calcium-targeted neuroprotection for IS. The schematic depicts the transition from preclinical promise to clinical failure, highlighting five critical mechanistic barriers. The potential efficacy of calcium modulators is often compromised by temporal delays relative to the hyperacute therapeutic window and dose-limiting toxicities. Furthermore, lack of target specificity, poor delivery across the BBB and microvascular no-reflow, and counterproductive systemic hemodynamic effects (e.g., cerebral steal phenomenon) collectively negate neuroprotective benefits in patient populations.

In summary, the repeated failures of past clinical trials do not refute the central pathological role of calcium overload; rather, they expose a systemic translational gap in applying single-target molecular interventions to the complex reality of human disease. Successful future strategies must holistically address multiple factors, including ultra-early therapeutic windows, precise cell- or pathway-specific targeting, optimized drug delivery systems capable of crossing the BBB, and minimization of systemic hemodynamic disturbances (Figure 3).

## 6. Expectations

Re-examining the molecular mechanism of calcium overload after ischemic stroke within the overall framework of spatiotemporal evolution and the interaction of neurovascular units is the cognitive starting point to break through the current transformation bottleneck. Calcium overload is a well-established pathogenic mechanism in ischemic stroke, and numerous high-quality reviews have provided in-depth molecular insights—including recent discussions on mitochondrial calcium regulation [69], neuronal calcium dyshomeostasis and therapeutic strategies [14], and systematic evaluations of long-term neuroprotective effects in animal models of calcium modulation [141] and so on. These contributions have laid a solid foundation for the field. However, the majority of these reviews remain confined within a “neuron-centric” and linear reductionist paradigm that emphasizes single targets at discrete time points. In contrast, our review offers a more comprehensive and up-to-date synthesis of the current understanding of calcium overload mechanisms in ischemic stroke. To the best of our knowledge, this represents the most exhaustive overview in the field to date, distinguished by several unique contributions and differentiated value: (i) integration of both preclinical and clinical trial status for calcium overload-targeted agents; (ii) comprehensive incorporation of the spatiotemporal dynamics of calcium overload; (iii) balanced coverage of both neuronal and non-neuronal cell types; and (iv) an updated, critical appraisal of calcium overload-directed interventional strategies. To critically situate the molecular machinery of stroke within a spatiotemporal context, future therapeutic strategies must transition from a “one-size-fits-all” approach toward precise temporal stratification and targeting innovation. In the temporal dimension, treatment should be rigorously phase-stratified:

Hyperacute phase (0–3 h): Alongside large-vessel recanalization, adjunctive microcirculatory protective strategies—such as targeting pericyte TMEM16A—are essential to ensure that blood flow effectively reaches the tissue.

Acute/subacute phase (3–24 h): Interventions should focus on anti-inflammatory and blood–brain barrier protective effects, for instance by inhibiting microglial P2X7/NLRP3 or endothelial Piezo1 to interrupt the self-perpetuating injury cycle.

Recovery phase (days to weeks): Strategies must pivot toward harnessing and modulating calcium signals (e.g., optogenetically enhancing astrocytic Ca^2+^ waves) to promote neuroplasticity and repair.

On the targeting dimension, strategies must evolve from crude “blockade” to refined modulation, guided by several core principles: (i) Development of subcellular organelle-specific agents (e.g., MCU inhibitors): Given the difficulty of globally controlling cytosolic Ca^2+^, it is more pragmatic to target downstream effectors. Inhibiting the mitochondrial calcium uniporter (MCU), for instance, prevents mitochondrial Ca^2+^ overload and ROS burst without perturbing physiological cytosolic Ca^2+^ signaling. (ii) Uncoupling strategies: Drawing inspiration from Nerinetide, pathological Ca^2+^ influx can be selectively disrupted by interfering with protein–protein interactions (e.g., assembly of the STIM1–Orai1 complex) rather than completely occluding the channel. (iii) Network pharmacology-driven multi-target combination regimens. The integration of this spatiotemporal framework with precision targeting represents a pivotal forward-looking direction for advancing the clinical translation of next-generation neuroprotective agents in stroke.

Furthermore, the field of calcium overload research is burdened by a long-overlooked structural limitation: the vast majority of preclinical studies remain confined to neuronal cultures or focal ischemia models in young, healthy animals. These models fail to recapitulate the remodeling characteristics of calcium signaling under comorbid conditions such as aging, hypertension, or diabetes, nor do they incorporate real-world clinical variables—including hemodynamic fluctuations and barriers to cerebral drug delivery.

Establishing a reverse translational research loop—spanning clinical phenotyping, mechanistic validation, and intervention optimization—is a critical step toward bridging the gap between preclinical and clinical evidence. Specifically, priority should be given to the following initiatives: (i) Reconstruction of calcium signaling networks based on human stroke biospecimens; (ii) Validation and stratification of candidate targets in animal models with comorbidities; (iii) Coordinated development of imaging biomarkers (e.g., calcium-sensitive MRI probes) and liquid biopsy tools (e.g., calmodulin in neuron-derived exosomes) to enable non-invasive, dynamic monitoring of calcium overload burden.

## 7. Conclusions

In conclusion, calcium overload in ischemic stroke is far more than a simple disruption of ionic equilibrium—it constitutes a highly complex, multicellular, and dynamically evolving systemic pathological network. The repeated translational failures of the past decades have laid bare a fundamental deficit in our understanding of the spatiotemporal dynamics that govern this intricate cascade. A paradigm shift is therefore imperative: we must move beyond the obsolete neuron-centric logic of wholesale calcium channel blockade toward a new framework defined by pan-neurovascular unit (NVU) coverage, whole-course temporal stratification, and multi-level precision modulation. Neurons do not operate in isolation; indeed, emerging evidence suggests that non-neuronal players—such as pericyte TMEM16A, endothelial Piezo1, and glial P2X7 receptors—may contribute even more substantially to the calcium overload cascade than conventional neuronal targets. This recognition mandates a strategic reorientation toward comprehensive NVU protection, with interventions targeting astrocytic STIM1, microglial NLRP3 inflammasomes, and pericyte TMEM16A channels representing particularly promising and translationally tractable directions. Critically, post-ischemic calcium signals are spatiotemporally heterogeneous: in the hyperacute phase, they manifest as physical microcirculatory obstruction; in the acute phase, as biochemical toxicity; and in the recovery phase, as regenerative cues. Therapeutic strategies must therefore be time-sensitive, achieving a dynamic equilibrium between suppression and modulation tailored to the evolving pathological context. Importantly, the failure of clinical candidates does not invalidate the calcium hypothesis—it instead exposes the bankruptcy of reductionist intervention logic built on single targets, disregard for hemodynamic compromise, and neglect of physiological signaling. The translational gap has been further widened by our collective underestimation of hemodynamic influences, cerebral drug delivery barriers (particularly microvascular no-reflow), and the dual roles of non-neuronal cells under comorbid conditions.

Future breakthroughs will hinge on uncoupling strategies that preserve physiological Ca^2+^ fluxes, nanotechnology-enabled delivery systems capable of bypassing BBB and no-reflow obstacles, and systems biology-guided multi-target combinations that address the network nature of calcium dysregulation. Only when preclinical research critically interrogates the limitations of isolated molecular targets and integrates profound clinical insights into microcirculatory failure, systemic inflammation, and patient heterogeneity can we hope to bridge the chasm between bench and bedside. The development of truly effective neuroprotective and reparative therapies for stroke patients will ultimately depend on our ability to master calcium as a “fire of life”—harnessing its regenerative potential while averting its destructive force, and transforming the treatment of ischemic stroke from a trial-and-error endeavor into a science-informed art of precision modulation.

## Figures and Tables

**Table 1:** Summary of Calcium Overload Mechanisms in Different Cells.

Cell Type	Target/Mechanism	Pathological Effect	Intervention Strategy
Neuron	NMDAR (GluN2B subtype): Excessive activation of glutamate → increased calcium influx.	Calcium overload → activates Calpain, CaMKII-DRP1, and TLK2 → mitochondrial division, nuclear membrane rupture, and apoptosis.	Telaprevir (inhibits GluN2B); Memantine (eNMDAR antagonist) EGCG (up-regulates hippocalcin to reduce calcium toxicity).
	TRPM2-esNMDAR coupling: Prolongs calcium influx.	Amplify excitotoxicity and exacerbate neuronal death.	TRPM2 inhibitors (ACA/TAT-M2).
Astrocyte	Downregulation of GLT-1: Disorder of glutamate reuptake.	Accumulation of glutamate in the synaptic cleave → prolongs neuronal excitotoxicity.	SHPL-49 (up-regulated GLT-1); s-GO reduces glutamic acid release.
	Calcium overload and ROS synergy: Oxygen-glucose deprivation/reoxygenation (OGD/R) induces injury.	Aggravate the destruction of NVUs.	tFNA inhibits calcium overload and ROS.
Microglial cell	TLR4/AKT/NF-κB pathway: Calcium influx activates the inflammatory response.	Release IL-6 and TNF-α → Disrupt the BBB; Recruit immune cells.	Inhibit the TLR4 pathway; The absence of CX3CR1 alleviates calcium influx.
	SOCE-mediated calcium overload: driving pathological activation.	Aggravate neuroinflammation and neuronal damage.	CRAC channel inhibitor
Vascular endothelial cell	Cavβ3 regulation: Affects tight junctions and BBB permeability.	Calcium signal disorder → disruption of the BBB → brain edema.	Target Cavβ3 to maintain BBB integrity.
	TRPM2 overactivation: Endothelial calcium overload.	Intensify the dysfunction of NVUs.	TRPM2 inhibitors

Abbreviation: BBB, blood–brain barrier. s-GO, small-sized graphene oxide. tFNA, Tetrapod framework nucleic acid. SOCE, store-operated calcium entry. NVUs, neurovascular units.

**Table 2:** Summary of Calcium Overload Mechanism and Targets.

Target/Mechanism	Pathological Effect	Intervention Strategy
Calpain system	Disrupt the ZO-1 of the BBB and increase permeability; Mediate the excessive activation of NMDA receptors; Cutting neuroprotective proteins such as TRPC6 and TrkB exacerbates neuronal death.	Calpeptin (a Calpain inhibitor) alleviates neuroinflammation and promotes vascular regeneration.
Ca^2+^/CaM-CaMKII-DRP1 pathway	Activating DRP1 phosphorylation induces excessive mitochondrial division, leading to energy metabolism disorders, increased ROS and neuronal apoptosis.	BAPTA-AM (calcium chelating agent) blocks the pathway and restores mitochondrial dynamic balance.
TLK2 kinase	TLK2 activation → nuclear membrane rupture and abnormal mitosis of neurons (mitotic disaster), and triggering apoptosis.	Promethazine (TLK2 inhibitor) or Blebbistatin (myosin IIA inhibitor) alleviates nuclear membrane rupture.
Wnt/Ca^2+^ signaling pathway	Activate PLC → IP_3_ → intracellular calcium release → CaMKII amplification → calpain activation, oxidative stress and apoptosis.	Gen down-regulates Wnt5a/Frizzled-2/p-CaMKII/IP_3_R; Upregulating SOD1/SOD2 alleviates oxidative damage.
PI3K/AKT pathway	Calcium overload inhibits PI3K/AKT, upregulates the ratio of Bax/Bcl-2, and activates apoptosis of CASP3.	CA activates PI3K/AKT, inhibits Bax and reduces the release of inflammatory factors.
Mitochondrial MCU gating imbalance	Reduction of calcium influx threshold; Insufficient phosphorylation of NCLX → mitochondrial calcium overload → inhibition of the respiratory chain, induction of mitochondrial ROS and mPTP opening.	Ru265 (MCU blocker); Bay 60-7550 (Activate PKA → Phosphorylate NCLX); Nanotechnology delivery (such as TAT/GYR peptide targeting mitochondria).
ER SERCA dysfunction	Decrease in SERCA pump activity → ER calcium depletion → cytoplasmic calcium accumulation → activation of UPR to promote apoptosis; Calcium flows to mitochondria → mitochondrial calcium overload.	RIPostC up-regulates the expression of SERCA2; SERCA2a gene therapy Neuroserpin inhibits the PERK-EIF2α pathway.
Glutamate-NMDA receptor	Glutamate accumulation in the synaptic cleft → excessive activation of the GluN2B subtype → calcium overload → mitochondrial dysfunction and apoptosis.	Telaprevir (inhibits phosphorylation of GluN2B); Memantine (eNMDAR antagonist) SHPL-49 (up-regulates GLT-1 in astrocytes and enhances glutamate clearance).
TRP channel (TRPM2/TRPV1)	TRPM2 activation → calcium overload in endothelial cells → disruption of the BBB; TRPV1 overactivation →Ca^2+^ -dependent cytotoxicity.	ACA/TAT-M2 peptide (TRPM2 inhibitor); KXA (down-regulating TRPV1). Hyperforin extract and resveratrol (inhibits TRPC6 degradation).
Energy metabolism disorders and membrane pump deactivation	Calcium overload → inhibition of oxidative phosphorylation → ATP depletion → inactivation of PMCA/SERCA/Na^+^/K^+^-ATPase → a vicious cycle of calcium overload.	Insulin pretreatment reduces calcium influx; Target mitochondrial energy metabolism (such as regulating NAD^+^ levels).

Abbreviation: BBB, blood–brain barrier. UPR, unfolded protein response.

**Table 3:** Comparison of preclinical and clinical outcomes of calcium-targeted intervention strategies in IS.

Category	Target/Strategy	Specific Drug/Example	Preclinical Findings	Clinical Outcomes and Key Reasons for Failure
Agents That Have Undergone Clinical Testing	L-type CCB	Nimodipine	Inhibits VGCCs and reduces reperfusion injury in animal models.	INWEST, VENUS and other trials showed no benefit or even worsened outcomes [104,113]. Key reasons: 1. Timing—delayed administration (typically >6 h) missed the critical window for early calcium overload. 2. Specificity—non-selective blockade interfered with normal neuronal signaling and cerebral autoregulation. 3. Side effects—systemic hypotension induced “steal phenomenon” in the ischemic penumbra, exacerbating injury.
	NMDA receptor antagonist	Aptiganel, Selfotel, Nelonemdaz	Blockade of NMDA receptor-mediated Ca^2+^ influx reduces infarct volume in animal models [114].	Aptiganel and Selfotel were terminated due to psychiatric side effects (hallucinations, agitation) and increased mortality. Nelonemdaz failed to improve outcome in the RODIN trial [115]. Key reasons: 1. Timing—clinical administration occurred after the glutamate peak. 2. Specificity—early agents non-selectively blocked both synaptic and esNMDARs, interfering with physiological survival signaling. 3. Side effects—severe neuropsychiatric toxicity.
	Magnesium ion	Magnesium Sulfate	Acts as a natural calcium antagonist; competitively inhibits NMDA receptors and MCU; neuroprotective in animal models.	Large-scale trials (FAST-MAG, IMAGES) did not improve functional outcomes [116,117]. Key reasons: 1. BBB penetration—polar molecule with limited ability to cross an intact BBB and reach therapeutic concentrations in brain parenchyma. 2. Timing—even ultra-early administration (e.g., FAST-MAG) may still miss the critical window of the molecular cascade.
	PSD-95 uncoupling agent	Nerinetide (NA-1)	Disrupts the NMDAR–PSD95–nNOS death signaling pathway, reducing infarct size without affecting physiological signaling in animal models.	ESCAPE-NA1 trial was neutral overall, but a beneficial effect was observed in the subgroup not treated with alteplase [118,119]. Key reasons:1. Drug interaction—plasmin generated during tPA thrombolysis degraded the peptide drug Nerinetide. 2. Specificity challenge—revealed unpredictable pharmacokinetics in complex clinical settings.
	Free radical scavenger	NXY-059	Attenuates oxidative stress and indirectly influences calcium-related injury in animal models.	SAINT II trial failed to meet its primary endpoint [120,121,122]. Key reason: Poor BBB penetration limited access to intracranial targets.
Novel Targets at Preclinical Stage	MCU inhibitor	Ru360, Ru265	Specifically blocks mitochondrial Ca^2+^ uptake, prevents mitochondrial Calcium overload, ROS burst and mPTP opening; protects neurons without affecting cytosolic Ca^2+^ signals in animal models [77,123,124].	Not yet entered clinical trials. Considered a promising next-generation neuroprotective strategy targeting “delayed calcium dysregulation”. Ru265 shows neuroprotective efficacy; pro-convulsant effects at higher doses warrant caution (lower doses appear safer).
	Astrocytic SOCE inhibitor	STIM1/Orai1 inhibitor (e.g., GSK-7975A [125])	Inhibits aberrant Ca^2+^ influx following ER store depletion in astrocytes; corrects reversal of glutamate transporters and attenuates excitotoxicity [32,126].	Not yet entered clinical trials. Targeting non-neuronal cells for indirect neuroprotection may avoid side effects of direct neuronal blockade. Main drawback: current inhibitors (e.g., GSK-7975A) lack sufficient selectivity, may partially affect Orai3 or other Ca^2+^ channels, leading to potential off-target effects and safety concerns.
	Pericyte TMEM16A inhibitor	Ani9	Inhibits ischemia-induced Ca^2+^-dependent chloride channel TMEM16A in pericytes; relieves capillary rigor and improves microvascular “no-reflow” without causing systemic hypotension [43,127].	Not yet entered clinical trials. Addresses a key clinical problem—post-recanalization microcirculatory failure—with a novel strategy. Main drawbacks: although Ani9 is relatively selective for TMEM16A, its in vivo pharmacokinetics (brain penetration, half-life, long-term safety) remain insufficiently optimized; lacks large-scale multicenter validation and human safety data.
	Endothelial Piezo1 inhibitor	Genetic interventions or small-molecule inhibitors	Blocks mechanosensitive Piezo1 channel; inhibits CaMKII/calpain pathway activation; preserves tight junctions; attenuates edema and hemorrhagic transformation [38,128].	Not yet entered clinical trials. Directly targets a key mechanism of BBB injury during reperfusion. Limitations: limited selectivity (not fully specific for Piezo1, may affect other mechanosensitive channels); insufficient brain penetration and long-term safety data; genetic approaches (e.g., knockout) are difficult to translate clinically; overall translational hurdles remain substantial.
	Microglial P2X7 receptor inhibitor	Various small-molecule inhibitors	Inhibits ATP–P2X7–NLRP3 inflammasome axis; reduces pro-inflammatory cytokine release and aberrant Ca^2+^ waves; attenuates neuroinflammation in animal models [129,130,131].	Not yet advanced to pivotal stroke trials. Major challenge: suppressing excessive inflammation without compromising microglial physiological surveillance and reparative functions.

## Data Availability

No new data were created or analyzed in this study. Data sharing is not applicable to this article.

## Abbreviations

Abbreviation	Full Term	Abbreviation	Full Term
2-APB	2-Aminoethoxyindophenyl Borate	BBB	Blood–Brain Barrier
CA	6′-O-Caffeyl Arbutin	cAMP	Cyclic Adenosine Monophosphate
CaM	Calmodulin	CaMKII	Ca2XCalmodulin-Dependent Protein Kinase II
CASP3	Caspase-3	CCB	Calcium Channel Blocker
CM-EM-137	CRAC Channel Inhibitor Compound	COX	Cytochrome c Oxidase
CRAC	Calcium Release-Activated Calcium Channel	CREB	cAMP Response Element-Binding Protein
CSD	Cortical Spreading Depolarization	CX3CR1	CX3C Chemokine Receptor 1
DAMPs	Damage-Associated Molecular Patterns	DRP1	Dynamin-Related Protein 1
EGCG	Epigallocatechin Gallate	EIF2α	Eukaryotic Initiation Factor 2 Alpha
ER	Endoplasmic Reticulum	ESCAPE-NA1	Endovascular Stroke Therapy Followed by Neures
esNMDAR	Extrasynaptic N-Methyl-D-Aspartate Receptor	FAST-MAG	Field Administration of Stroke Therapy—Magn
GABA	Gamma-Aminobutyric Acid	Gen	Genistein
GLT-1	Glutamate Transporter-1	GluN2A	N-Methyl-D-Aspartate Receptor Subunit 2A
GluN2B	N-Methyl-D-Aspartate Receptor Subunit 2B	GPCR	G Protein-Coupled Receptor
GRP75	Glucose-Regulated Protein 75	GSK3β	Glycogen Synthase Kinase 3 Beta
GsMTx4	Grammostola spatulata Mechanotoxin 4	GYR	Glycine-Arginine-Tyrosine Peptide
IL-1β	Interleukin 1 Beta	iNOS	Inducible Nitric Oxide Synthase
INWEST	Intravenous Nimodipine West European Stroke	IS	Ischemic Stroke
JNK	c-Jun N-Terminal Kinase	KXA	Kuaxiong Aerosol
MALT1	Mucosa-Associated Lymphoid Tissue Lymphom	MAMs	Mitochondria-Associated Membranes
MCU	Mitochondrial Calcium Uniporter	mGluR	Metabotropic Glutamate Receptor
MICU1	Mitochondrial Calcium Uptake 1	mPTP	Mitochondrial Permeability Transition Pore
NCLX	NaXCa2XExchanger	NIHSS	National Institutes of Health Stroke Scale
NMDAR	N-Methyl-D-Aspartate Receptor	NO	Nitric Oxide
NOX	NADPH Oxidase	nNOS	Neuronal Nitric Oxide Synthase
NVU	Neurovascular Unit	OGD/R	Oxygen-Glucose Deprivation/Reoxygenation
Orai1	Calcium Release-Activated Calcium Channel Pre	P2X7	Purineric Receptor P2X7
PDE2	Phosphodiesterase 2	PERK	Protein Kinase R-Like Endoplasmic Reticulum F
Piezo1	Piezo-Type Mechanosensitive Ion Channel Comp	PKA	Protein Kinase A
PLA_2_	Phospholipase A2	PMCA	Plasma Membrane Ca2XATPase
PMZ	Promethazine	RIPostC	Remote Ischemic Postconditioning
RODIN	Randomized Study of Nelonenadaz for Ischemic	ROS	Reactive Oxygen Species
Ru265	Ruthenium-Based MCU Inhibitor	SAINT II	Stroke-Acute Ischemic NXY Treatment II Trial
SERCA	Sarco/Endoplasmic Reticulum Ca2XATPase	SOD1	Superoxide Dismutase 1
SOD2	Superoxide Dismutase 2	SOCE	Store-Operated Calcium Entry
STM1	Stromal Interaction Molecule 1	TAT	Trans-Activator of Transcription Peptide
t-PA	Tissue Plasminogen Activator	TLK2	Tousled-Like Kinase 2
TLR4	Toll-Like Receptor 4	TNF-α	Tumor Necrosis Factor Alpha
TrkB	Tropomyosin Receptor Kinase B	TRP	Transient Receptor Potential
TRPC6	Transient Receptor Potential Canonical 6	TRPM2	Transient Receptor Potential Melastatin 2
TRPM4	Transient Receptor Potential Melastatin 4	TRPV4	Transient Receptor Potential Vanilloid 4
UPR	Unfolded Protein Response	VDAC	Voltage-Dependent Anion Channel
VENUS	Very Early Nimodipine Use in Stroke Trial	VGCC	Voltage-Gated Calcium Channel
ZO-1	Zonula Occludens-1		
